# Diverse Response to Local Pharmacological Blockade of Sirt1 Cleavage in Age-Induced versus Trauma-Induced Osteoarthritis Female Mice

**DOI:** 10.3390/biom14010081

**Published:** 2024-01-08

**Authors:** Yonathan H. Maatuf, Miya Marco, Shani Unger-Gelman, Eli Farhat, Anna Zobrab, Ankita Roy, Ashish Kumar, Idan Carmon, Eli Reich, Mona Dvir-Ginzberg

**Affiliations:** Institute of Biomedical and Oral research, Faculty of Dental Medicine, Hebrew University of Jerusalem, P.O. Box 12272, Jerusalem 9112102, Israel; yonatan.maatuf@mail.huji.ac.il (Y.H.M.); miya.marco@mail.huji.ac.il (M.M.); shani.unger@mail.huji.ac.il (S.U.-G.); eli.farhat@mail.huji.ac.il (E.F.); anna.zobrab@mail.huji.ac.il (A.Z.); ankita.roy@mail.huji.ac.il (A.R.); ashish.kumar1@mail.huji.ac.il (A.K.); idan.carmon@mail.huji.ac.il (I.C.); reich.eli@gmail.com (E.R.)

**Keywords:** Sirt1, aging, intra-articular, osteoarthritis, senescence

## Abstract

**Objective**: Previous studies have shown that the cleavage of Sirt1 contributes to the development of osteoarthritis (OA). In fact, OA was effectively abrogated by the intra-articular (IA) administration of two compounds, one blocking Sirt1 cleavage (CA074me) and the other activating Sirt1 (SRT1720), using a post-traumatically induced model (PTOA) in young female mice. In this study, we attempted to understand if this local treatment is effective in preventing age-associated OA (AOA) progression and symptoms. **Design**: A group of 17-month-old female C57BL/6J mice were IA administered with CA074me and/or SRT1720 or their combination. Joint histopathological analysis and bone histomorphometry were carried out, with an assessment of knee mechanical hyperalgesia. A serum analysis for NT/CT Sirt1 was carried out along with immunohistochemistry for articular cartilage to detect p16^INK4A^ or γH2A.X. Similarly, meniscal cartilage was monitored for Lef1 and Col1a1 deposition. The data were compared for young female mice subjected to post-traumatic OA (PTOA). **Results**: Similar to PTOA, combination-treated AOA exhibited improved knee hyperalgesia, yet structural improvements were undetected, corresponding to unchanged NT/CT Sirt1 serum levels. Both AOA and PTOA exhibited unchanged staining for nuclear p16^INK4A^ or γH2A.X and lacked a correlation with OA severity. Contrarily to PTOA, the combination treatment with AOA did not exhibit a local reduction in the Lef1 and Col1 targets. **Conclusions**: When targeting Sirt1 cleavage, the PTOA and AOA models exhibited a similar pain response to the combination treatment; however, they displayed diverse structural outcomes for joint-related damage, related to Lef1-dependent signaling. Interestingly, nuclear p16^INK4A^ was unaffected in both models, regardless of the treatment’s effectiveness. Finally, these findings highlight the variations in the responses between two highly researched OA preclinical models, reflecting OA pathophysiology heterogeneity and variations in gender-related drug-response mechanisms.

## 1. Introduction

Aging is accompanied by a dramatic increase in the prevalence of degenerative diseases, and it has emerged as one of the most urgent biomedical and socio-economic challenges facing developed countries [1]. Many of these age-related diseases share similar signaling pathways and ultimately result in increased cellular senescence, an irreversible halt in cell division [2]. Cellular senescence increases with aging and correlates with progressive tissue degeneration and the appearance of numerous age-related comorbidities. One such condition is the progressive joint degenerative disease, osteoarthritis (OA), which is highly prevalent with age and associated with cellular senescence [2].

It has been established that senescent cells occur more frequently during the aging process [2] and that they emerge in articular tissue after trauma or injury [3]. As such, there may be an additional link between cellular senescence and trauma-related acute inflammation, potentially a key contributor to increasing the volume of senescent cells during OA pathogenesis. Conversely, senescent cells contribute to a systemic increase in inflammatory mediators [4] through their capacity to secrete a host of cytokines, collectively termed senescence-associated secretory phenotype (SASP) exosomes. This suggests that age-related senescence can influence a plethora of systemic effects, including immune cell senescence [5], neuroendocrine circuits, such as thyroid function [6], and various inflammatory growth factors and hormones [7].

Amongst some of the established aging markers, SIRT1 activity has been extensively reported to promote longevity and genomic stability, in part by the targeted deacetylation of two central factors: the p65/RelA subunit of NFκB, which influences inflammation, and p53, which influences cell cycle progression [8,9]. The enzymatically active SIRT1 can remove p53 acetylation and divert the fate of damaged cells from cellular senescence toward apoptosis. Nonetheless, environmental pro-inflammatory insults may contribute to the loss of SIRT1 activity, in part by the cathepsin-B-mediated cleavage of its C-terminal domain [10]. In previous studies, we have identified fragments of serum derived from the early stages of OA in preclinical and clinical cohorts (i.e., NT/CT SIRT1) [11]. Notably, the emergence of NT/CT Sirt1 was related to accumulated p16^INK4A^ staining in mouse articular cartilage, and both markers were impaired when applying senomorphic therapies in a systemic and local route for a preclinical aging/ACLT model [11]. 

In an attempt to block SIRT1 cleavage to activate it effectively, we intra-articularly (IA) administered two compounds (i.e., CA074me blocking cathepsin B and/or SRT1720 activating Sirt1, noted as a “combination treatment”) in a post-traumatically induced OA (PTOA) young female mouse model [12]. As a result of administering the combination treatment, we reported reduced pain, hyperalgesia, and structural damage following PTOA [12]. These results were mechanistically associated with a reduction in chondrocyte Lef1 nuclear activity and meniscal Col1a1 deposition [12]; however, an association between the combination treatment and p16^INK4A^ articular chondro-senescence was not established. Here, we attempted to assess whether p16^INK4A^ articular chondro-senescence is affected by the combination treatment and determine if the same IA treatment administered to the PTOA model, has similar beneficial effects in an age-induced spontaneous OA model (AOA) for female mice of the same background (i.e., C57BL/6J). Notably, spontaneous OA has been displayed in mice of the same background over the age of 17 months [13,14]. Thus, we chose the same temporal administration and extent employed in PTOA model [12], for AOA (i.e., 17-month-old mice) and assessed the pain, structural outcomes, and cellular mechanisms targeted by this treatment, based on our previous reports suggesting chondro-senescence [11] or regulation mediated by Lef1 and its targets [12,15].

## 2. Materials and Methods

### 2.1. Mice Experimental Procedures

Mice-related experimental procedures were carried out in accordance with NIH Committees for Animal Use and Care (ARAC guidelines), based on the Association for Assessment and Accreditation of Laboratory Animal Care International (AAALAC) guidelines, and in accordance with the ARRIVE guidelines. The Hebrew University Institutional Animal Care and Use Committee approved the study’s protocols (AOA; MD-22-16752-4 and PTOA; MD-12-13383-4). Mice were subjected to 12 h light/dark cycles and received food and water ad libitum.

C57BL/6J (Jackson lab, Bar Harbor, ME, USA) WT mice (females, n ≥ 4) were aged to reach 17 months and were thereafter administered intra-articularly (IA) twice a week for 4 weeks as follows: vehicle (20v% DMSO in PBS), Sirt1 activator (i.e., SRT1720; 81 μM in 5 μL; ApexBio, Houston, TX, USA, cat#: A8239), cathepsin B inhibitor (CA074me; 600 μM in 5 μL; BioVision, Milpitas, CA, USA, cat#: 2772), or a combination of the Sirt1 activator and cathepsin B inhibitor (concentrations as indicated above, overall 10 μL volume). After 4 weeks of IA treatment, the mice were euthanized by cervical dislocation following anesthesia (ketamine and xylazine 200 mg/kg). Joints were collected for histological analysis, as detailed below. Local joint pain measurements were carried out using a pressure applicator measurement device (PAM, Ugo Basile, Gemonio, Italy) at two timepoints during the experiment: (a) before the beginning of the IA injection regime and (b) prior to euthanizing the mice. During the PAM procedure, the mice were restrained by the operator, who simultaneously applied pressure to the mouse joint using a special force sensor on the operator’s thumb. After the force was applied, this elicited the animal response (i.e., limb withdrawal), which was automatically recorded. Each measurement consisted of triplicate measurements for each mouse, with 1-minute breaks between measurements. Measurements were taken following mouse habituation a week prior to the experiment, partially carried out with a blinded experimenter.

Similarly, the female PTOA experiments were carried out by surgical destabilization of the medial meniscus (DMM) procedure, as previously described [12,16]. These female mice (n = 7) were locally administered with the compounds as indicated above after 4 weeks of surgical PTOA procedures. Structural, histological, and pain phenotypes related to these mice were previously published in Elayyan et al., 2022 [12], and partial data from this publication were adapted herein for comparison. C57BL/6J (Jackson lab, Bar Harbor, ME, USA) WT male mice aged 16.5 months (n = 5) were sacrificed and used as the controls for immunofluorescent staining. Mice aged 16–18 months are referred to as AOA mice.

### 2.2. Histology and Immunohistochemistry

Mouse tibiofemoral joint cartilage samples were processed for histology as previously reported [12]. Briefly, the samples were fixed for 3 days in 4% formaldehyde and decalcified for 21 days in 10% EDTA at pH = 7.5 (which was replenished every 3 days). The samples were dehydrated in a graded series of ethanol washes before being embedded in paraffin and sectioned to 7 μm slices.

For OA histopathology, joint sections were stained with 0.5% Safranin O (cat# 1.15948, Merck, Kenilworth, NJ, USA) and 0.1% Fast green (cat# 1.04022, Merck-Millipore, Burlington, MA, USA), following staining with Weigert’s Iron Hematoxylin (Merck-Millipore, Burlington, MA, USA cat# 1.15973). The stained sections were visualized under light microscopy. OARSI histopathology grading was carried out by at least three blinded referees, as previously reported [12].

Osteophyte formation was monitored on a 0–3 scale (with 3 denoting the most severe case) as previously described [12]. Additionally, the degree of synovitis was determined based on the synovial thickness and appearance of F4/80-positive staining within the synovial membrane area. Briefly, the synovial lining was stained by H&E staining (Mayer’s Hematoxylin, cat# 1100, Kaltek, Saonara, Italy; Eosin Y, cat# 3801601E, Leica Biosystems, Deer Park, IL, USA), and the membrane region was extracted manually from each joint image to be subsequently uploaded to the ImageJ software (version: 1.52a). Using the ImageJ processing tools, a number of colored pixels were converted to mm^2^ to indicate the thickness of the selected inner synovial membrane area.

For immunohistochemistry (IHC), sections were processed as previously described [11,12]. Briefly, the sections were digested with 1 mg/mL hyaluronidase (Sigma-Aldrich, St. Louis, MO, USA, cat# H3506) in PBS at pH 6 for 1 h at RT and stained with rat anti-mouse F4/80 (cat#: MCA497GA, Bio-Rad Laboratories Ltd., Haifa, Israel), rabbit anti-mouse Col1a1 (1:250, cat# ab21286, Abcam, Cambridge, UK), or mouse anti-p16^INK4A^ (1:500; cat#: ab54210; Abcam, Cambridge, UK) primary antibody. HRP goat anti-rat antibody (1:2000, cat#: ab97057, abcam plc., Waltham, MA, USA), goat anti-rabbit (1:300; ab7090, abcam plc., Waltham, MA, USA), and goat anti-mouse antibody (1:1000; cat#: ab97040, abcam plc., Waltham, MA, USA), respectively, were used as the secondary antibodies. The negative controls were incubated with a secondary antibody alone and counterstained with hematoxylin. Secondly, IHC of F4/80+ macrophages or p16^INK4A^ cells (brown) was carried out and quantified by the ImageJ:IHC_Toolbox.jar plugin per analyzed area for F4/80 or identified chondrocyte nuclei stained with p16^INK4A^ beyond a threshold of 120 intensity (in arbitrary units or AU). Notably, F4/80+ macrophages were detected in the inner synovial membrane of the joint, while nuclear p16^INK4A^ was detected in the articular cartilage tissue. Col1a1 deposition was quantified for the inner tip of the lateral meniscal cartilage (Figure 7D illustration).

For immunofluorescence, the sections were rehydrated with decreasing concentrations of ethanol, incubated for 1 h with a blocking solution containing 0.1% BSA, 1:10 FBS, and 1:50 Triton X100 in PBS, and subjected to room-temperature (RT) conditions, as previously described [12]. The sections were incubated with rabbit Anti-Lef1 antibody (1:150, cat# ab137872; Abcam) or Phospho-Histone H2A.X (Ser139), denoted γH2AX (1:500, cat# a #2577; Cell Signaling Technology, Danvers, MA, USA), overnight at 4 °C and thereafter incubated with Alexa flour 568 anti-rabbit (1:1000, #cat A-11011, abcam plc., Waltham, MA, USA) for 2 h at RT. After antibody staining, the sections were incubated with DAPI (final 5 ng/μL in DDW) for 10 min at RT, washed four times with PBS, and mounted (IMMUMOUNT, Thermo Fisher, Waltham, MA, USA). The slides were captured using a Nikon Eclipse Ti Fluorescence microscope (Nikon, Tokyo, Japan) equipped with a Photometrics Prime 95B camera (Teledyne Photometrics, Tucson, AZ, USA), and the images were analyzed using the NIS-Elements AR analysis software (Nikon Instruments Inc., Tokyo, Japan, version: 5.11.01). The quantification of nuclear Lef1 was carried out for the inner tip of the lateral meniscal cartilage (as shown in Figure 7D illustration), while all tibiofemoral articular chondrocytes positive for γH2AX and DAPI co-appearance (threshold intensity of 645 AU) were recorded per total DAPI-stained nuclei in each field.

### 2.3. Micro Computed Tomography (μCT) Analysis

Mouse joints were scanned using a Skyscan 1272 scanner (Bruker Inc., Kontich, Belgium) with the following parameters: voltage (60 kVp), current (150 μA), time of exposure (845 ms), filter (0.25), aluminum resolution (2016 × 1344), pixel size (6.4 μm), and degree of rotation (0.4 degrees). Scanned images were reconstructed using the NRecon software (Bruker Inc., Belgium, version: 2.1.0.2) with a beam hardening correction of 70% and a ring artifact correction of 11%. The reconstructed data were analyzed using the CTAn software (Bruker Inc., Belgium, version: 1.21.2.0+). Different ROIs were chosen for the various joint compartments that were measured: 30 slices from the anterior meniscus, 50 slices for the tibial subchondral cortical plate, and the tibial trabecular bone volume under the subchondral plate. The sampling location for the cortical plate and trabecular bone volume was the intercondylar eminence of the joint.

### 2.4. Detection of NT/CT Sirt1 Levels in Mice Serum

The serum from the experimental end stages was collected and diluted (1:2000) for indirect ELISA assays using a standard curve ranging from 0.2 to 100 ng/mL full-length human or mouse SIRT1 proteins, according to the procedure by Batshon et al. [12]. Diluted serum was incubated with a C-terminus reactive antibody (anti-mouse SIRT1 #2028, Cell Signaling Technology, Danvers, MA, USA) or an N-terminus reactive antibody (anti-human/mouse SIRT1, cat# 07-131, Merck-Millipore, Burlington, MA, USA; polyclonal antibody corresponding to amino acid 1–131 of human and mouse NT SIRT1). The values were compared to the standard curve, and an NT/CT Sirt1 ratio was calculated. As previously shown, an increase in the NT/CT Sirt1 ratio in mice was reflective of early OA and chondro-senescence [11].

### 2.5. Statistical Analysis

A statistical analysis was carried out using two- or one-way ANOVA tests for mean rank comparisons, considering *p* < 0.05 to be statistically significant. Subsequent paired comparisons were carried out using the Mann–Whitney test between the two treatment groups. Spearman’s correlation was carried out for p16^INK4A^ staining and OA severity, assuming an r rank of 1 to be the strongest correlation. Plots and statistical analysis were carried out using the Prism software (version 9, GraphPad Software, Boston, MA, USA). Schematic images were generated using the BioRender software (https://www.biorender.com (accessed on 4 January 2024), Toronto, ON, Canada).

## 3. Results

### 3.1. p16^INK4A^ Is Unaffected by Combination Treatment in Young PTOA Mice

**Figure 1:**
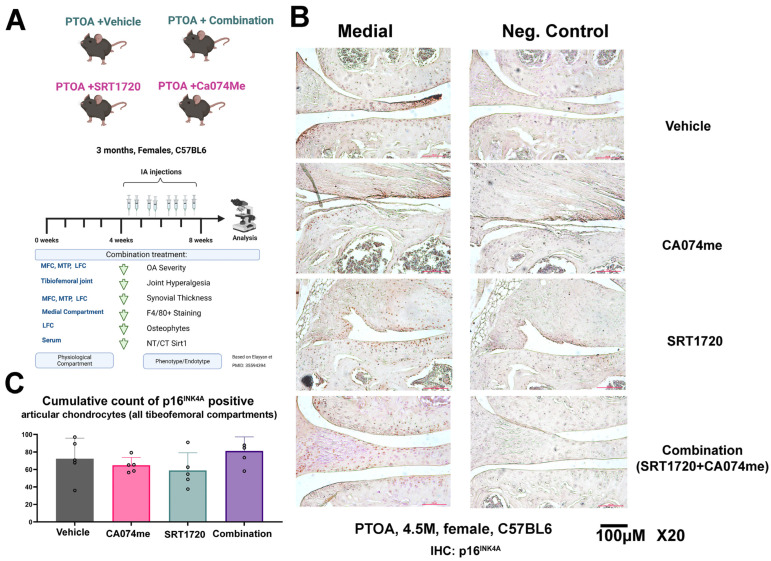
Post-traumatic female mice treated with combination drugs exhibiting unchanged p16^INK4A^ levels. (**A**) PTOA experimental setup adapted from Elayyan et al., 2022 [12], denoting the structural outcomes of the vehicle, CA074me-, SRT1720-, and combination-treated PTOA female mice (3 months of PTOA induction; treatment from 4 weeks of PTOA; and sacrifice after 8 weeks of PTOA). (**B**) Representative immunohistochemistry for p16^INK4A^ of PTOA vehicle (n = 5), SRT1720 (n = 5)-, CA074Me (n = 5)-, and combination (n = 4)-treated female mice (3 months) depicting the medial articular compartment and a negative control for p16^INK4A^ staining (denoted “control”) at a ×20 magnification. (**C**) The graphs display nuclear p16^INK4A^ intensity beyond a threshold of 120, as judged by ImageJ software analysis. Accumulated positive p16^INK4A^-stained nuclei within articular cartilage tissue within all four tibiofemoral compartments (i.e., medial tibial, medial femoral, lateral tibial, and lateral femoral) are plotted and presented per treatment group (n ≥ 4). Statistical significance was determined using a one-way ANOVA test among each experimental group, with *p* < 0.05.

In a previous study, we used a surgical procedure wherein mice underwent PTOA via destabilization of the medial meniscus (DMM), as previously described [16]. Using 3-month-old female mice treated with the combination treatment for 4 weeks, PTOA resulted in improved pain, hyperalgesia structural improvements in the cartilage, and reduced synovitis (Figure 1A scheme) [12]. Given the fact that Sirt1 cleavage or a loss of activity were previously shown to be associated with chondro-senescence [11], we herein tested whether the combination treatment was able to reduce p16^INK4A^, which is considered one of the cellular hallmarks of a senescent phenotype [17]. Notably, the action of p16^INK4A^ on cell-cycle-regulating cyclins is predominantly nuclear [17], which is supported by reports displaying an increased nuclear intensity for p16^INK4A^ associated with senescence in the context of kidney disease [18]. Therefore, we assumed that p16^INK4A^ nuclear staining indicates cartilage senescence and damage. Contrary to our expectations, PTOA treated with a combination did not display any changes in nuclear p16^INK4A^ staining vs. the vehicle control (Figure 1B,C). These data indicate that the combination treatment does not mechanistically target p16^INK4A^ when preventing OA-related structural damage. Interestingly, other researchers have also shown that p16^INK4A^ is not a sufficient hallmark to determine structural joint damage in mice [19].

### 3.2. Local Intra-Articular Administration of Combination Treatment to AOA Improves Pain Hyperalgesia without a Structural Effect

**Figure 2:**
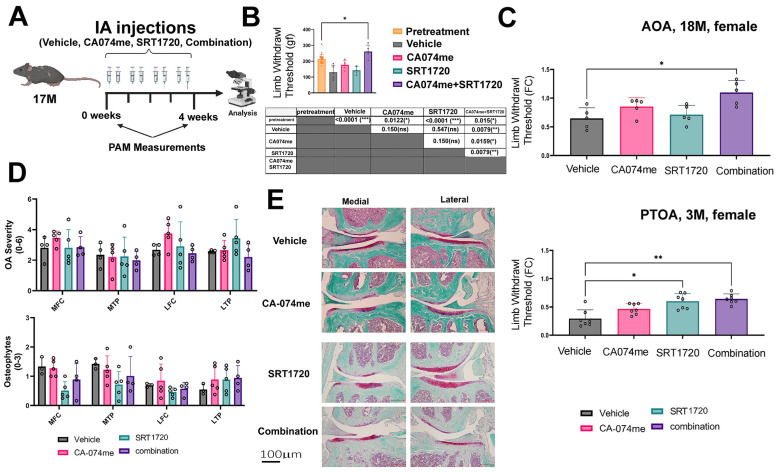
Intra-articular combination treatment improves hyperalgesia without affecting articular cartilage in AOA female mice. (**A**) Schematic experimental array: female WT mice were aged to 17 months and subjected to intra-articular injections twice a week for 4 weeks, as previously reported by Elayyan et al. [12]. The pain threshold was measured before the onset of the injection and before sacrifice via PAM applied to the joint. (**B**) Pain measurement of AOA for injected female WT mice. The table below the graph shows the *p*-values for all paired treatments. (**C**) This denotes paired PAM ratios to a given mouse treatment vs. baseline in fold change (FC) for AOA and PTOA, the latter adapted from Elayyan et al., 2022 [12]. (**D**) Assessment of articular cartilage for OA severity (range 0–6; top) and osteophyte formation (range 0–3; bottom). Medial femoral condyle denoted “MFC”; medial tibial plateau denoted “MTP”; lateral femoral condyle denoted “LFC”; lateral tibial plateau denoted “LTP”. (**E**) Representative depictions of Safranin-O-/Fast-green-stained section of the tibiofemoral joint, with a ×20 magnification. Statistical significance was determined using a two- (**D**) or one-way (**B**,**C**) ANOVA test for experimental groups, with *p* < 0.05, followed by a Mann–Whitney test, between two treatment groups, assuming *p* < 0.05 to be statistically significant (panel **B**,**C**). * for *p* < 0.05, ** for *p* < 0.01, **** for *p* < 0.0001.

The experimental setting employed herein included 17-month-old female mice (C57BL/6J) administered IA with CA074me (a cathepsin B inhibitor), SRT1720 (a specific Sirt1 activator), or a combination of the two drugs vs. a vehicle control (Figure 2A). The results show that the knee withdrawal thresholds were lower in the vehicle, CA074me, or SRT1720 alone vs. baseline; however, a higher pain threshold was observed in the combination treatment vs. all other treatments, including the baseline pain levels denoted in the following graph and table (Figure 2B). An additional paired analysis per mouse was carried out to determine the fold change (FC in plots) in pain vs. baseline of a given mouse for AOA and PTOA models (Figure 2C). The data show a consistent increase in the fold change threshold for AOA and PTOA, with the latter adapted from the data in Elayyan et al., 2022 [12].

### 3.3. Local Intra-Articular Administration of Combination Treatment to AOA Did Not Elicit Structural Changes in Joint Tissues

**Figure 3:**
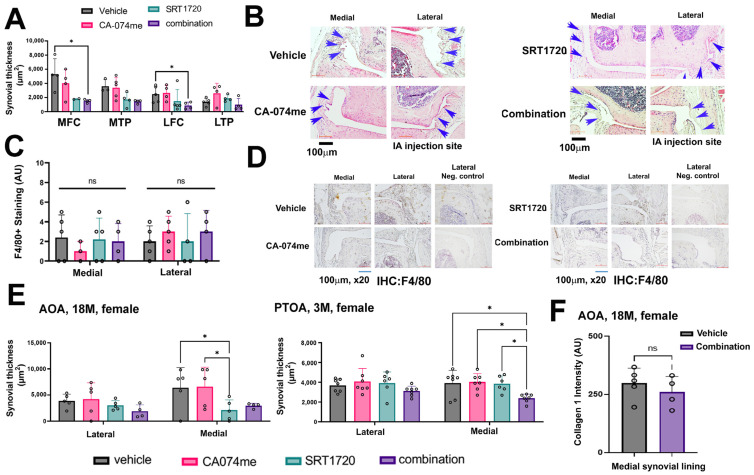
Analysis of AOA synovium displayed reduced thickness with undetected changes in F4/80 staining. (**A**) AOA joints were taken for the assessment of synovial thickness following H&E staining and ImageJ software analysis of the inner synovial lining. Combination treatment exhibited reduced synovial thickness in the femoral compartments of joints. Medial femoral condyle denoted “MFC”; medial tibial plateau denoted “MTP”; lateral femoral condyle denoted “LFC”; lateral tibial plateau denoted “LTP”. (**B**) Representative images of the synovium of the injected mice marked with blue arrows for synovial cell lining thickness (AOA). (**C**) Quantification of IHC staining for F4/80 in the inner synovial tissue. (**D**) Displays representative images of F4/80-stained synovium and a negative control from the lateral compartment (denoted “Lateral Neg. control”) for AOA. (**E**) Cumulated synovial thickness for lateral or medial compartments of AOA and PTOA female mice. PTOA data in right graph adapted from Elayyan et al., 2022 [12]. (**F**) Collagen type 1 staining for the medial synovial lining of AOA mice subjected to IA combination vs. vehicle control. Statistical significance was determined using a two-way ANOVA test for experimental groups with *p* < 0.05, followed by a Mann–Whitney test (panel **A**,**E**,**F**), between two treatment groups, assuming *p* < 0.05 to be statistically significant. * for *p* < 0.05, “ns” for not significant.

Contrary to pain phenotypes, when assessing OA severity and osteophyte formation, the experimental groups did not display structural changes (Figure 2D,E). Further assessment of the mouse joints for synovitis determined that there was a significant reduction in the synovial thickness between the vehicle treatment and the combination treatment for the femoral compartment of the joints (Figure 3A,B), with undetected changes in F4/80+ staining (Figure 3C,D). An analysis of the lateral and medial synovial thickness for AOA and PTOA exhibited a medial response in both models, potentially due to the lateral IA administration mode, which may have punctured the synovial lining to a significant extent (Figure 3E). Accordingly, we monitored the unpunctured medial compartment, which displayed reduced synovial thickness (right plot, Figure 3E) for combination-treated PTOA, in line with Elayyan et al., 2022 [12]. However, the combination treatment in AOA did not exhibit a statistically significant reduction for the medial compartment treated with the combination treatment (left plot, Figure 3E). Further, collagen 1 staining for the AOA combination treatment vs. vehicle displayed unchanged levels (Figure 3F), implying that the changes in the synovial thickness as a result of the combination treatment may not be a result of synovial tissue fibrosis.

**Figure 4:**
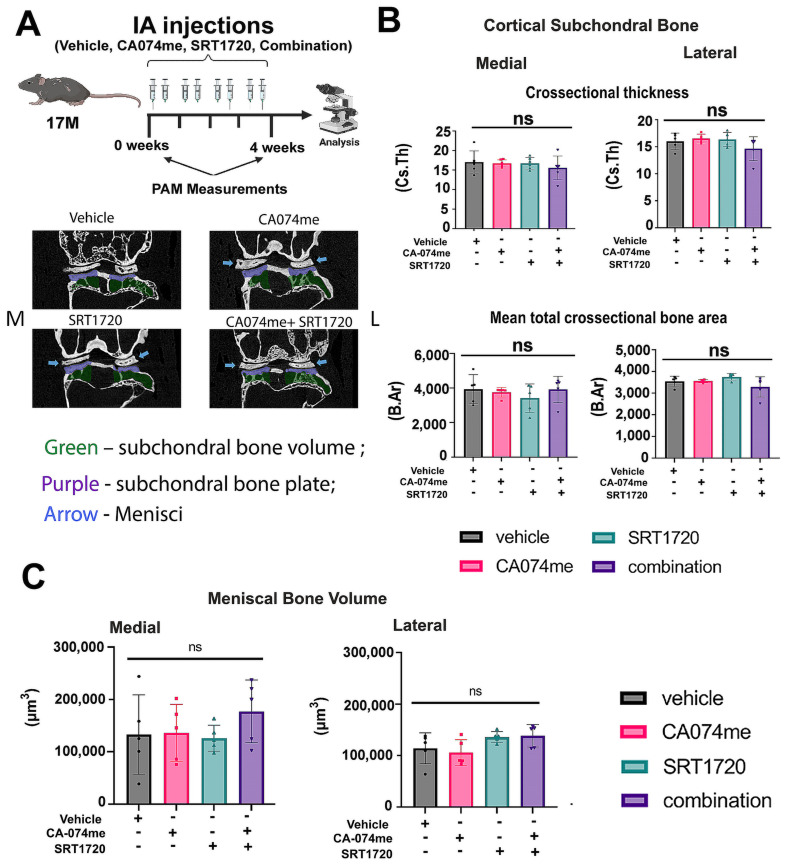
Combination treatment does not impact joint bone structural characteristics for AOA female mice, as judged by μCT analysis. Tibiofemoral joints were scanned via μCT for (**A**) subchondral bone plate cross-sectional thickness (Cs.Th) and Mean total crossectional bone area (B.Ar) (**B**), and meniscal volume (**C**) of 17-month-old female WT mice treated with SRT1720 and/or CA074me (n ≥ 4). Statistical significance was determined using a two-way ANOVA test for experimental groups, with *p* < 0.05. “ns” for not significant.

Analysis of the joint bone morphometry (Figure 4A), including the subchondral tibial bone plate (Figure 4B) and meniscal mineralization (Figure 4C), did not reveal any changes between the four AOA treatment groups.

### 3.4. Local Intra-Articular Administration of Combination Treatment to AOA Did Not Alter the NT/CT Sirt1 Ratio or Nuclear p16^INK4A^ Chondrocyte Staining

**Figure 5:**
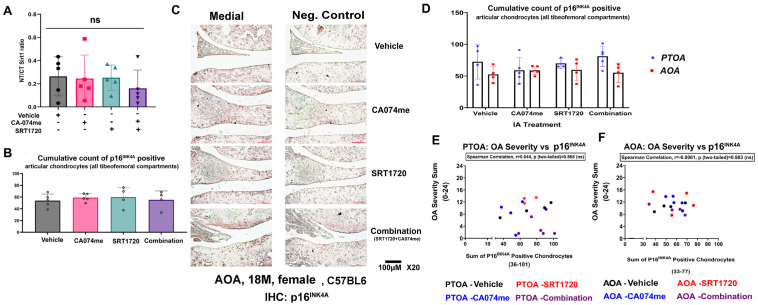
AOA mice did not exhibit changes in serum NT/CT Sirt1 and cartilage nuclear staining for p16^INK4A^: (**A**) Serum NT/CT Sirt1 levels were measured using the indirect ELISA method. (**B**) Graphs depict quantification of nuclear p16^INK4A^ immunohistochemistry in articular cartilage. (**C**) Representative images of p16^INK4A^-stained (**left**) and -negative control (**right,** denoted “Neg. Control”) from AOA vehicle (n = 5), SRT1720 (n = 4), CA074me (n = 5), and combination (n = 4). Medial articular compartments are presented at a magnification of X20. (**D**) Cumulative p16^INK4A^ nuclear staining of all tibiofemoral compartments for PTOA and AOA treatment groups, with no statistical significance between the groups according to a two-way ANOVA test. Correlation of p16^INK4A^ immunohistochemistry and OA severity from (**E**) PTOA or (**F**) AOA was plotted and assessed using Spearman’s correlation (black dots: vehicle; blue dots: CA074me; red dots: SRT1720; and purple dots: combination). Spearman’s correlation r value and two-tailed value are displayed above, with each plot signifying no correlation between p16^INK4A^ and OA severity for both groups (i.e., AOA and PTOA). PTOA data in panel E were adapted from Elayyan et al., 2022 [12]. “ns” denotes not significant.

We next attempted to assess whether NT/CT Sirt1 fragments were altered with the treatment, as we previously found a significant reduction in the marker for PTOA following a combination treatment in female mice [12] (SI_15B in report). The data in Figure 5A do not show variations in the serum NT/CT Sirt1 fragments, which also corresponds with the undetected changes in OA-related joint structure between the groups (Figure 2D). Similarly, nuclear p16^INK4A^ staining remained unchanged between the treatment groups (Figure 5B,C). Moreover, when comparing the four treatment groups of PTOA and AOA, we could not detect differences in the p16^INK4A^ nuclear intensity (Figure 5D). While it is possible that 4 weeks after PTOA resulted in p16^INK4A^ accumulation, the lack of any change between the vehicle PTOA and AOA for p16^INK4A^ implies that it may not be a significant contributor to articular cartilage damage in female mice, regardless of the treatment’s efficacy. Finally, it should be noted that the limited AOA group, comprising 4–5 mice, could limit our conclusions.

**Figure 6:**
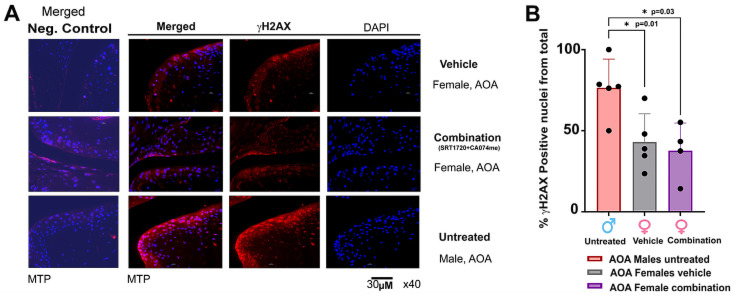
Male untreated AOA mice exhibit higher γH2AX levels in articular cartilage than vehicle- or combination-treated female AOA mice. (**A**) Immunofluorescent staining for % γH2AX-positive nuclei from total nuclei was measured for articular compartments of 16.5-month-old male untreated (n = 5), female vehicle-treated (IA, n = 5), and female combination (IA, n = 4)-treated mice. Medial articular compartment is presented at a magnification of ×40. (**B**) Graph depicts quantification of % γH2AX-positive nuclei. Statistical significance was determined using a one-way ANOVA test for experimental groups, followed by a Mann–Whitney test, assuming statistical significance at *p* < 0.05.

Notably, both the AOA and PTOA treatment groups did not show any correlation between OA severity and p16^INK4A^ nuclear staining (Figure 5E,F) for all four treatments plotted in the separate graphs (i.e. PTOA or AOA). These data suggest that the combination treatment may not target p16^INK4A^ or that this marker does not necessarily reflect chondro-senescence and/or cartilage destruction related to senescence, which is similar to the results of other reports [19]. To further validate other markers of cellular senescence, we tested for γH2AX-positive nuclei in the articular cartilage for the AOA females treated with vehicle or combination, which were compared to untreated 16.5-month-old males (Figure 6A). The quantification of γH2AX-positive nuclei exhibited higher levels in the untreated males vs. the females, with no change amongst the treated female groups (Figure 6B), indicating that male mice may be more likely basally affected by age-related chondro-senescence.

### 3.5. AOA Models Treated with Combination Do Not Display Changes in Sirt1/Lef1-Related Mechanisms

**Figure 7:**
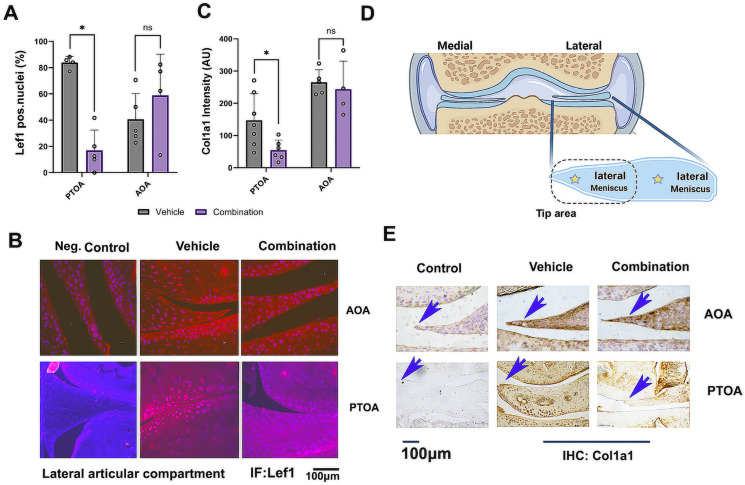
Combination treatment for AOA did not alter Lef1 nuclear staining: AOA mice were subjected to Lef1 immunofluorescence and quantified for the lateral joint compartment, similar to our previous report for PTOA young mice (graph adapted from Elayyan et al., [12]). (**A**) This displays a plot quantifying the percentage of positive cells for DAPI and Lef1 co-appearance. (**B**) Representative immunofluorescent images of AOA vehicle (n = 5), SRT1720 (n = 4), CA074me (n = 5), and combination (n = 4) are presented at a ×40 magnification. (**C**) Quantification of collagen type 1a1 (Col1a1) staining; (**D**) illustrates the region of interest for Lef1 and Col1a1 quantification of the inner “tip area” of the lateral meniscus. (**E**) Col1a1 representative images with blue arrows pointing at the quantified region, displayed at a magnification of ×40. Statistical significance was determined using a two-way ANOVA test for experimental groups (panels **A**,**C**), with *p* < 0.05, followed by a Mann–Whitney test (panels **A**,**C**) between two treatment groups, assuming *p* < 0.05 to be statistically significant. PTOA data in panels A and C were adapted from Elayyan et al., 2022 [12]. * for *p* < 0.05, NS for not significant.

Previously, we found that PTOA treated with combination therapy impaired Lef1 nuclear localization, resulting in lesser Col1a1 deposition [12] (shown in SI-11). Here, we attempted to determine whether this mechanism may be ineffectively targeted in the AOA model. Indeed, we found that the same dose of the combination treatment, did not reduce Lef1 nuclear staining in the AOA mice, while the opposite result was observed for the young female PTOA mice (Figure 7A–C,E); PTOA adapted from Elayyan et al., [12]).

Furthermore, we assessed the levels of meniscal Col1a1, which were reduced in the external lateral meniscus (Figure 7C,E illustration) of the PTOA mice treated with the combination treatment vs. vehicle ([12], SI-11). Here, too, we did not find an effect on the staining intensity of Col1a1 for the AOA-combination-treated mice vs. vehicle (Figure 7C,E, PTOA adapted from [12]).

Cumulatively, the data suggest that using a combination treatment to block Sirt1 cleavage and induce its deacetylase activity, cannot achieve the same structural results in the aging female joint compared to the younger PTOA female joint, which was reflected in the NT/CT Sirt1 serum biomarker and the histopathological assays. Moreover, the data suggest that this dose and the administration route were ineffective in targeting Lef1/Col1a1 in the AOA model, unlike the PTOA results. Finally, we found that p16^INK4A^ nuclear staining is likely irrelevant to the cellular circuits targeted by a combination treatment or insufficient for determining the chondro-senescence-related structural fluctuations that are characteristic of OA. One important consistent outcome from this study is that the combination treatment elicits mechanical allodynia for both PTOA and AOA female models, which may not be linked to synovial inflammation.

## 4. Discussion

The data herein insinuate that for AOA, we were unable to achieve structural alterations of the joint, using this dosage and local treatment. At least for this target, higher doses of the combination treatment may have exhibited more structural potency. Notably, these findings contrast with our previous reports using the same strain and sex of mice in a PTOA model [12]. Moreover, using a cannabinoid agonist administered systemically to old mice for a similar duration enabled us to improve the joint structure [14], indicating that AOA could present structural improvements in similar conditions of administration; however, this may change depending on the target.

In a well-designed study by Loeser et al. [20], 12-week-old (young) and 12-month-old (old) mice underwent PTOA induction via DMM and displayed a more severe structural phenotype in the old mice group, despite having the same insult, which indicates the possible involvement of age-associated systemic variables. Moreover, monitoring the contralateral (non-operated) joint of the old post-traumatic mice exhibited more significant structural joint deteriorations than the contralateral joint of the age-matched old sham group. On the other hand, no significant changes in OA severity between the young DMM and the sham contralateral joint were detected [20]. It is therefore evident that the mechanism of age-related OA pathology in preclinical models is more complex than that of post-traumatically induced OA mice. With age, many confounding systemic effects may contribute to the deterioration of the joint and its tissues. Numerous systemic changes may contribute to age-related OA pathophysiology, including age-related estrogen depletion [21], decreased IGF1 levels [22], and factors associated with metabolic syndrome [23], etc. Notably, aging mice models often exhibit similar knee hyperalgesia between sexes, which does not necessarily translate into the same degree of structural deterioration and progression [24].

Importantly, the lack of change in p16^INK4A^ in the female AOA and PTOA mice concurs with the notion that p16^INK4A^ may not necessarily be related to the degree of joint damage, as previously shown in [19], which is, according to our work here, relevant to preclinical drug-administered models. Indeed, while p16^INK4A^ is a key marker of senescence, there are many others, such as β-galactosidase, p21, and markers of genomic instability, such as SASP, which are also known to be related to a senescent phenotype [25]. It is impossible to assess senescence using one marker, and specialized markers toward particular tissues and conditions that involve senescence should be better characterized and tailored toward conditions and model. For example, channels that can regulate ectopic mineralization of the joint could be potential candidates to link the particular structural OA attributes (i.e., osteophyte formation) to the chondro-senescent phenotype [26]. While we detected p16^INK4A^ in all tissues regardless of the treatment’s efficacy, we observed that meniscal Lef1/Col1a1 was responsive to treatment in PTOA and not in AOA, confirming their downstream effects to Sirt1 activity in cartilage. Interestingly, a report by Azazmeh et al. showed that the chronic expression of p16^INK4A^ in epidermal tissue could elicit disease via WNT signaling activation [27], for which Lef1 is a critical nuclear mediator. Hence, the addition of WNT signaling constituents to the chondro-senescent signature may be relevant to OA development.

Notably, a significant difference between this work and others assessing p16^INK4A^ in C57BL/6J mice strains [3,19] is that, here, we exclusively used female C57BL/6J mice for administering the combination drug treatment, while other studies often used male mice. The use of male mice is often the case in OA, given that male mice develop severe and more progressive OA compared to female mice [28]. Our data, highlights that when assessing senescence, one should consider possible gender variations that can affect this pathway and potentially provide insight concerning gender-related pathogenesis involving senescence. For example, kidney disease development was reported to structurally vary between males and females, which is accompanied by variations in the expression of senescent markers in the various kidney zones [29].

Our data did exhibit an improvement in pain phenotypes for the combination treatment, which was not accompanied by changes in the levels of synovial macrophages; however, we did observe a reduction trend for synovial thickness in AOA similar to that of PTOA, when examining the unpunctured medial compartment. Interestingly, PTOA did exhibit a medial reduction in synovial thickness for the combination-treated young female mice, in tandem with reduced F4/80 staining [12], which was not the case for AOA. Given that changes in synovial thickness without alterations in synovial macrophage staining may be related to synovial fibrosis [30], we further assessed the staining of collagen 1 in the medial inner synovial lining, which remained unchanged in the AOA model. Notably, these data contradict previous reports indicating that synovial fibrosis is detected in painful OA patient subsets [31], as well as in preclinical rodent models [32]. Zhang et al. reported the highest collagen type 1 levels in synovium with DMM vs. ACLT, MIA, or the normal joint. In fact, this study reported that Col1a1 levels, indicative of synovial fibrosis, were negatively correlated with sensory innervation or the expression of markers related to pain sensation (i.e., CGRP, TRPV1, and NGF) in the PTOA models [32]. However, AOA models, and specifically, female mice, could potentially have varied pain-transmitted mechanisms that remain poorly defined.

Cumulatively, at least for the pharmacological blockade of Sirt1 cleavage, the data suggest that PTOA responds more aberrantly than AOA models and that senescence appears to be less affected in female mice in response to this treatment. As such, a particular treatment should be tailored to a specific model and should consider gender-related effects. These data are highly relevant when designing clinical trials toward the translation of potential DMOADs.

**Study limitations**: The AOA study was relatively limited given the small animal group used comprising 4–5 female mice. Moreover, we did not include sham controls for PTOA or younger mice for AOA, which limits the study conclusions. While including such control groups is of great importance, we attempted here to focus on the four treatment groups and their mechanistic and structural impact on joint tissues and OA pathogenesis.

**Study conclusions:**

o A diverse structural response to the same Sirt1 targeting treatments is apparent between AOA and PTOA female mice.
o Both AOA and PTOA are responsive to combination treatments for knee mechanical hyperalgesia.
o AOA mice treated with the combination showed an improved pain response but no structural improvement vs. the vehicle control.
o Treatment of the Sirt1 combination in AOA models does not result in variations in the Lef1/Col1 axis, which was not the case with PTOA in previous works [12].
o Combination treatment for PTOA or AOA does not target chondro-senescence in female mice.

## Data Availability

Data are contained within the article.
